# Agricultural, domestic and handicraft folk uses of plants in the Tyrrhenian sector of Basilicata (Italy)

**DOI:** 10.1186/1746-4269-1-2

**Published:** 2005-07-29

**Authors:** Giovanni Salerno, Paolo Maria Guarrera, Giulia Caneva

**Affiliations:** 1 Dipartimento di Biologia, Università di Roma Tre, Viale Marconi 446, 00146 Rome, Italy; 2 Museo Nazionale Arti e Tradizioni Popolari, Piazza Marconi 8/10, 00144 Rome, Italy

## Abstract

**Background:**

Research was carried out into agricultural and domestic-handicraft uses in folk traditions in the Tyrrhenian sector of the Basilicata region (southern Italy), as it is typically representative of ethnobotanical applications in the Mediterranean area. From the point of view of furnishing a botanical support for the study of local "material culture" data was collected through field interviews of 49 informants, most of whom were farmers.

**Results:**

The taxa cited are 60, belonging to 32 botanical families, of which 18 are employed for agricultural uses and 51 for domestic-handicraft folk uses. Data show a diffuse use of plants for many purposes, both in agricultural (present uses 14%; past uses 1%) and for domestic-handicraft use (present uses 40%; past uses 45%); most of the latter are now in decline.

**Conclusion:**

60 data look uncommon or typical of the places studied. Some domestic-handicraft folk uses are typical of southern Italy (e.g. the use of *Ampelodesmos mauritanicus *for making ties, ropes, torches, baskets or that of *Acer neapolitanum *for several uses). Other uses (e.g. that of *Inula viscosa *and *Calamintha nepeta *for peculiar brooms, and of *Origanum heracleoticum *for dyeing wool red) are previously unpublished.

## Background

Ethnobotanical studies supporting the ethno-anthropological sciences, and of "material culture", which describe aspects of farmers' and shepherds' economy now on the point of disappearance are infrequent in Italy. Generic news is sometimes present in botanical texts concerning, for example, handicraft uses, but the plant matter of single artefacts is rarely defined; this specific matter can change from place to place and can originate peculiar local artefacts. Ethnobotanical uses of plants are often lost more easily in modern civilisation, due to industrial activity that substitutes traditional handicrafts. This study was carried out in an area, the Tyrrhenian sector of Basilicata, where, in remarkable contrast with that happens in the rest of Italy, we can still witness a large folk employment of plants and a rich and intense memory of their uses is present. Due to history, economy and tradition, this area could potentially be a precious source of information already lost in other places.

A previous study of flora, vegetation and spontaneous food plants [1] underlined the still low industrial and urban impact upon the area of study, considered a good source of information for ethnobotanical applications typical in the central Mediterranean area.

This new ethnobotanical study was therefore carried out in order to document folk agricultural and domestic-handicraft usages, now on the point of disappearance, and with a view to observing potential new economic utilizations. This enquiry aims to fill a gap concerning the employment of useful plants in a territory of southern Italy, characterised in the past by the presence and influence of cultures of various origin (first and foremost among which is that of the Greek colonists).

**Figure 1:**
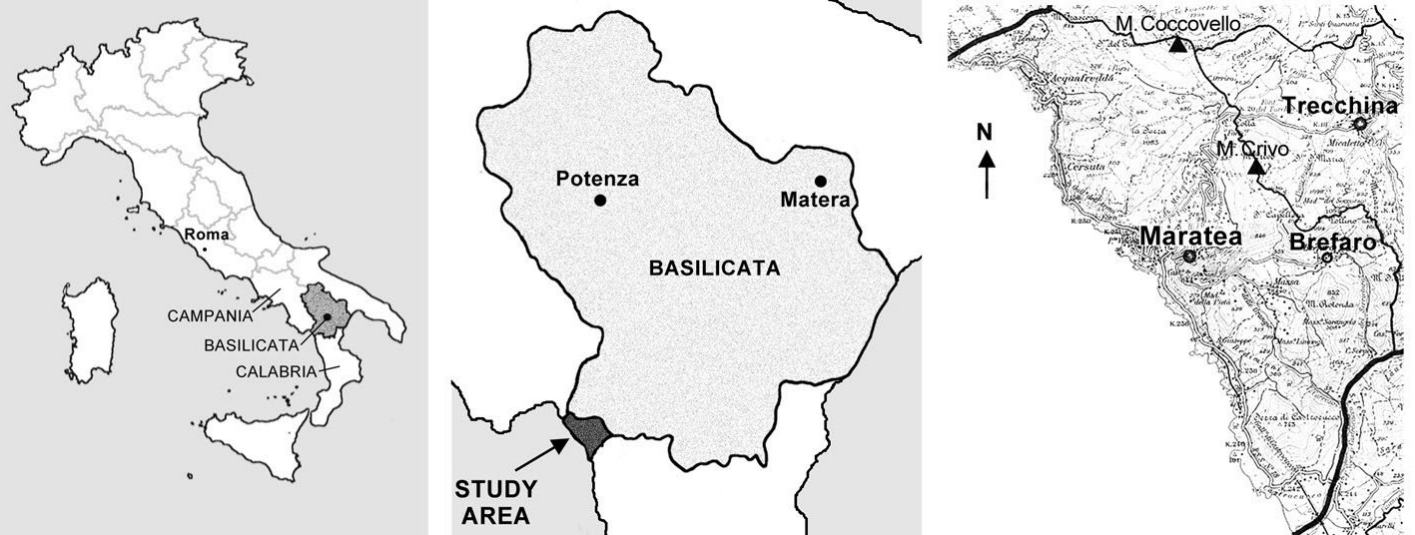
Geographical location of the field research area in the Basilicata region, Italy.

The research was carried out in the territory of Maratea (province of Potenza), with some scattered nuclei stretching from the coast to the slopes of Mt. S. Biagio (Massa, Brefaro) and of Trécchina, in the interior (total population 7600 inhabitants). It is located between the Campania region to the north and Calabria to the south (Fig. 1), and includes 28 km of indented coastline running from the Policastro Gulf to the plain of the Noce River. More than 70% of the territory is characterised by steep slopes and includes a calcareous and calcareous-limestone mountain ridge; the highest point is Mt. Coccovello (1505 m). The climate is of Mesomediterranean type, with rainfall concentrated in the period between October and March, with the arid months being limited to June and July. Precipitation is abundant, about 1200 mm of rainfall per year [2]. The average temperature in the coldest month (January) is about 8.02°C and the warmest month (August) about 27.9°C, with an annual average of 14°C. Roughly 1/3 of the land is suitable for agricultural use whilst, in the past, this quantity was increased by the use of terracing. Some areas are cultivated uniquely with olives and vines; other areas show a typical cultivation ("vignale") made by a mosaic of olives, vines and fruit trees. There are also arable lands and pastures. Mediterranean maquis and forests are present in the area, but also mixed woods, garrigues, shrubby areas, steppes and pastures [2,3].

## Methods

Field data were collected during the periods April – July 2002 and March 2003. Ethnobotanical data, mostly regarding the uses of wild plants was collected using structured interviews (some folk uses of cultivated plants are also reported). The informants were people who had been living in the research area for many years. The informants interviewed were 49 (26 men, 23 women) whose ages ranged from 25 to 94, and who belonged (mainly) to families with strong links to the traditional activities of the area. Most of the interviewees (34) were more than 50 years old, of whom: 5 between 50 and 60, 11 between 60 and 70, 15 between 70 and 80, and 3 over 90 years old. Among the informants 19 were farmers, the others shepherds, building workers, restaurateurs, housewives. Interviews were carried out using fresh plant specimens, or by visiting meadows and woods with the informants to collect the plants related to folk uses. Voucher specimens were kept in the Herbarium of the University of Rome Tre. Some tape recordings are also conserved in the National Museum of Arts and Folk Traditions of Rome. In the structured interviews (whose results are reported in Table 1, Additional file 1) informants were requested to indicate, for each plant: vernacular name, folk uses (in agricultural and domestic or handicraft fields), parts used, periods of gathering, frequency or rarity in the area of a given use according to the informants, and also to specify whether the use was personal (that is practised by the informant) and/or familiar (that is practised by one or more members of that family) and/or practised by others (e.g. acquaintances). Further information, related to possible personal consumption or sale of the substance or object produced, was requested. In table 1, Additional file 1 we also indicate the citations for each use (number of informants) and the current use (indicated with °). The nomenclature of the listed species follows Pignatti [4], but we have also used Tutin *et al*. [5] in the classification of the plants.

## Results and discussion

**Figure 2:**
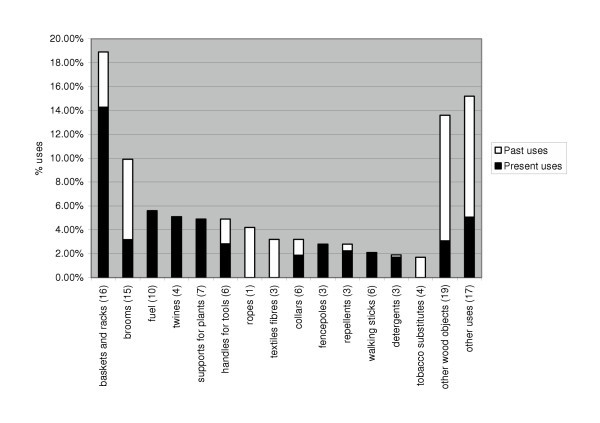
Main typologies of agricultural and domestic-handicraft uses, according to the number of citations, in the Tyrrhenian sector of Basilicata. For each category the number of species is indicated.

The plants of ethnobotanical use, as collected from the field study, are reported in Table 1, which summarise all original data from interviews and further controls in the field. The reported uses are 162. Most of the information are coincident with what was previously reported in the specific Italian consulted literature [6-16]; 56 data are however uncommon or typical of the places studied. Data show a diffuse use of plants for many purposes (Fig. 2), both in agricultural (present uses 14%; past uses 1 %) and for domestic or handicraft use (present uses 40%; past uses 45%); most of the latter are now in decline. The most meaningful and interesting uses are listed below.

### A) Agricultural uses

We found 18 wild species used in supporting agricultural activities: mostly as twine and "tutore", but also as shelter from winter frosts, as twines, as graftholders, or in 'sovescio' (green manure) activities.

**Figure 3:**
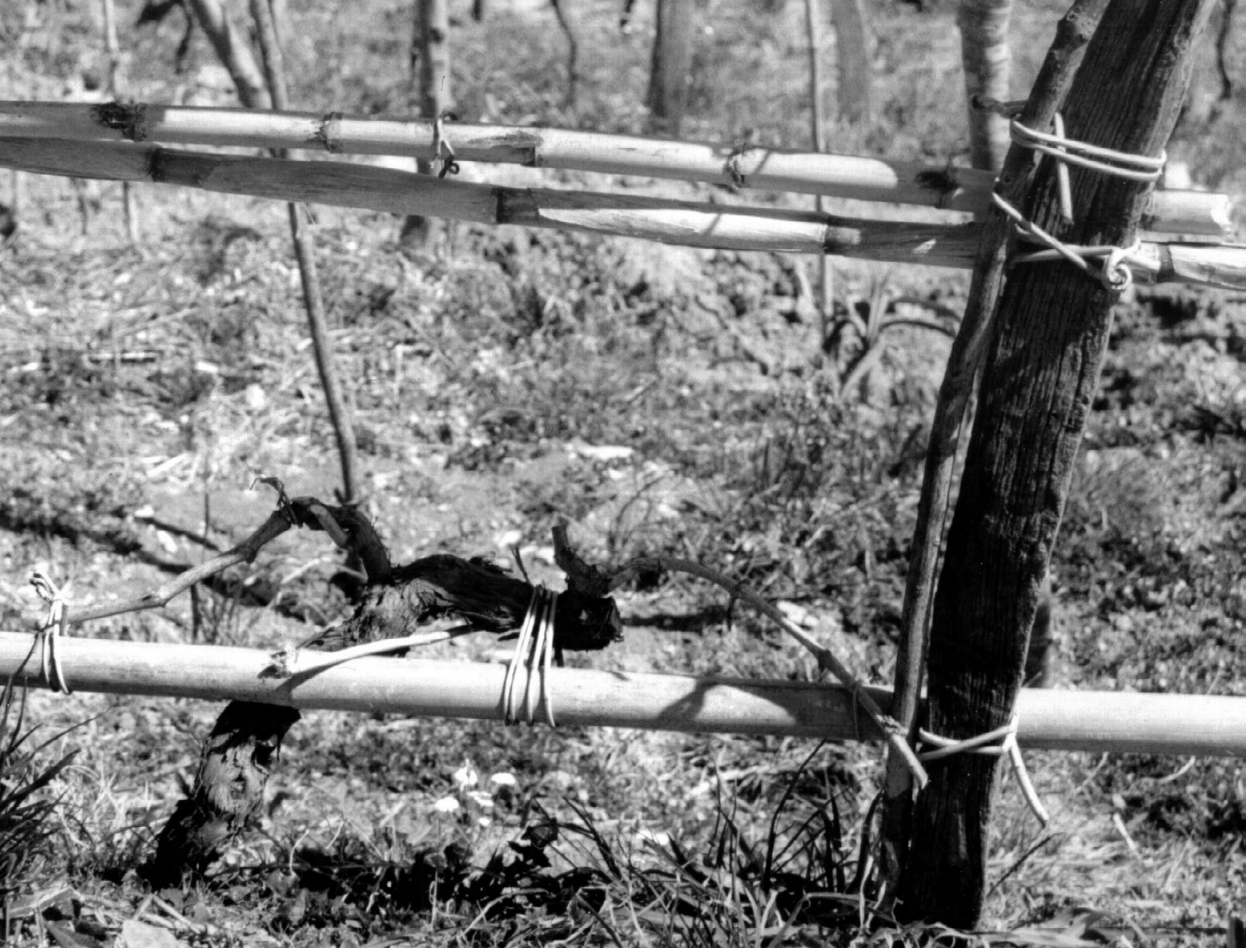
Vine tied with *Salix alba *subsp. *vitellina *branches.

We found many plant species used to fix cultivated plants to "tutori"; characteristic and relevant is the use of different species to support different parts of the plant, and in different seasons. For example, in the cultivation of the vine, in winter the branches that are supposed to product the fruit-bearing shoots are fixed to the "tutore" by using *Salix alba *subsp. *vitellina *(Fig. 3). The latter is planted along the edges of the whole vineyard and it is preferred for its long, thin and particularly flexible branches [7,17]; these features are obtained with a yearly pollarding. In spring-summer, on the contrary, the budding vine shoots are tied with *Ampelodesmos mauritanicus *leaves, to prevent their being broken by the weight of the bunches. The practice of tying vine with *Ampelodesmos *[9,11] is extremely old: the Greek name, in fact, literally means "twine for vine" (*ampelos *= vine, *desmòs *= twine). In the past, the plant was also gathered for sale in the neighbouring village, where it is less frequent. Other plants to cite are *Holoschoenus australis *and *S. junceum*, used above all as twine for vegetables [7,9,18]. The custom of using vegetal twines is disappearing, and with it we are losing the knowledge of the highly specific techniques of knotting.

One activity noted in Maratea in spring, is the use *Pyrus amygdaliformis *as a graft-holder for cultivated varieties of pear tree (in effect, it is the root of this pear tree that is used, since it is more robust and hardier). The practice is also recorded for Sicily [11] and Cilento (G. Salerno, unpublished data). All the uses cited are still practised in the territory; on the contrary, today the practice of using "green" manure (produced from a wide range of *Leguminosae*, e.g. *Vicia *sp. pl. and *Lathyrus sylvestris*) appears to be almost quite obsolete. The resumption of such a practice would be desirable as it could considerably limit the use of synthetic fertilizers. For example "tutore" are stakes, or longitudinally cut trunks, or even (unusual local use) stems not deprived of secondary ramifications to support creeping plants (e.g peas). The stems with leaves of *Quercus ilex*, *Arundo donax *and *Spartium junceum *are used to protect delicate cultivated plants from winter frosts (above all *Citrus*-trees), by creating a kind of folding screen around them.

### B) Domestic and handicraft uses

**Figure 4:**
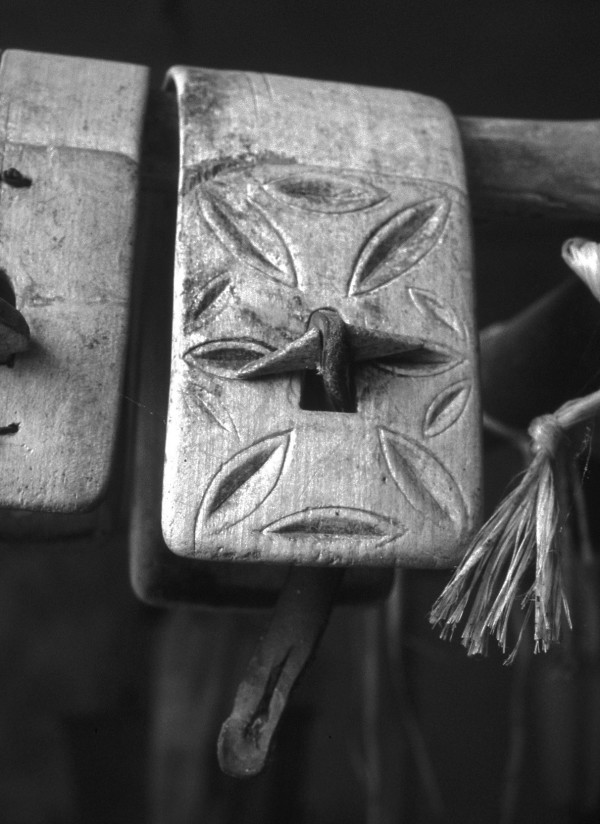
Decorations on a "puntagliera", special collar made with *Acer *sp. for goats and cows (various species of this genus are used to do such collars).

**Figure 5:**
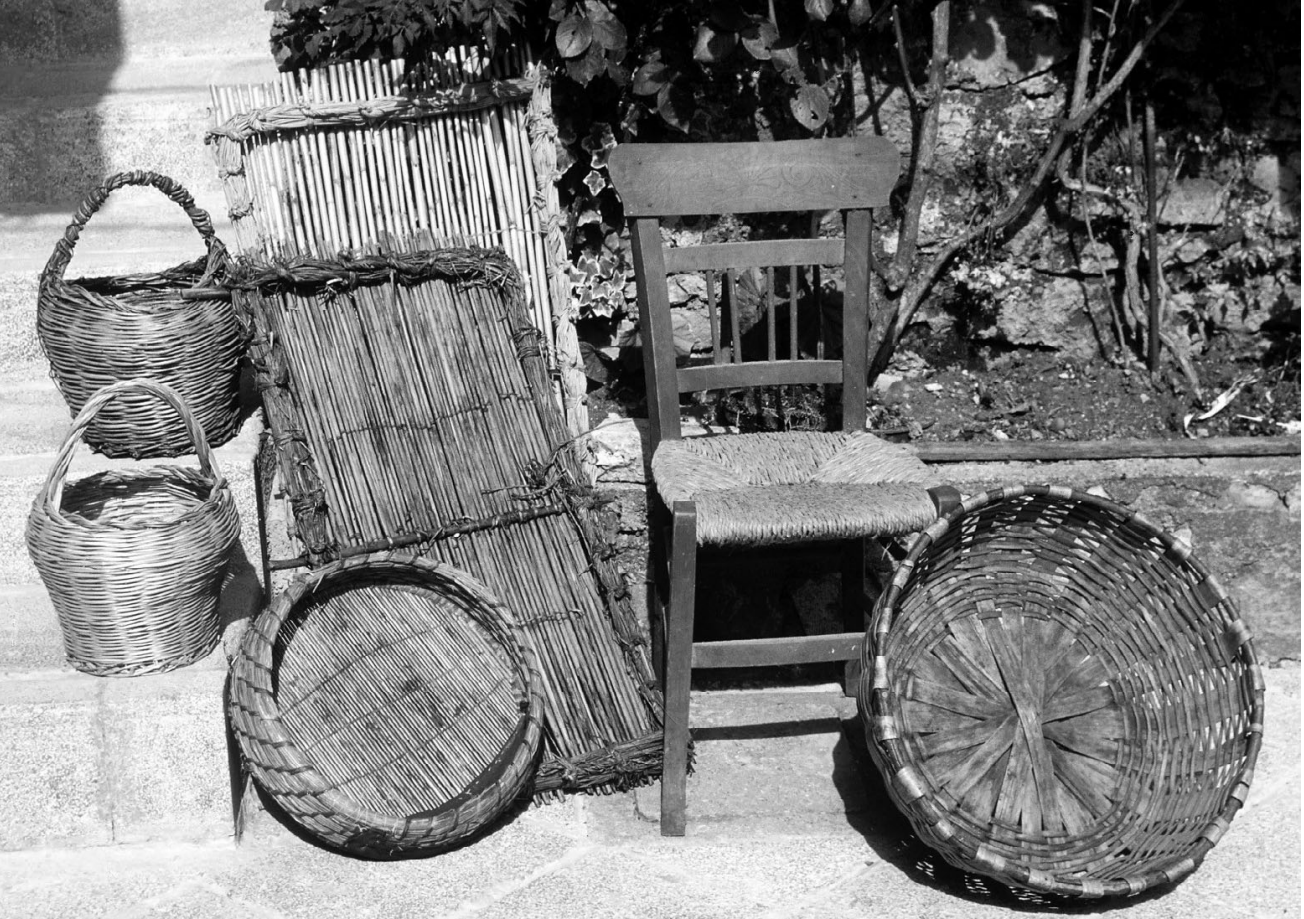
From left to right: baskets made with *Salix *sp. pl., sieve ("cernicchio") and rack ("gratedde") with *Ampelodesmos mauritanicus, Arundo donax *and *Spartium junceum*, chair with *Typha latifolia *and basket made with *Castanea sativa *and *Fraxinus ornus*.

**Figure 6:**
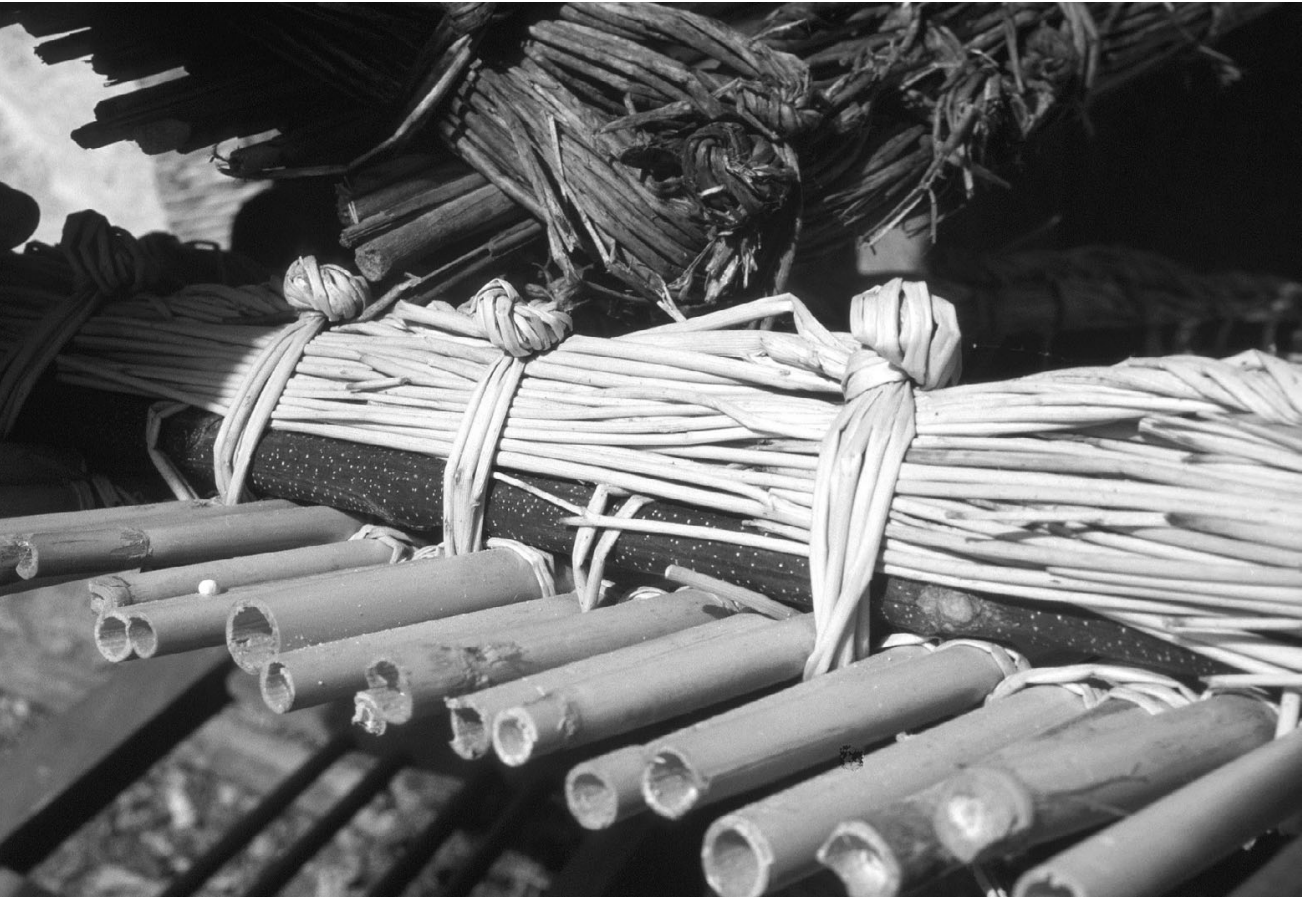
Rack ("gratedda"), tray made with *Arundo donax *and edge of *Spartium junceum*, used for drying foodstuffs.

In Maratea a remarkable number of plants (51) employed in local handicrafts or for domestic uses was recorded, in many cases involving the production of interesting and typical artefacts (Fig. 4, 5, 6).

A very high number of informants (36) were registered with regard to *A. mauritanicus*; a large proportion of the people of Maratea were involved in gathering its leaves [19]. Up until the 1960's, this plant had represented one of their main sources of income. Large faggots of the species were sold near the port, where the fibre was extracted from small bunches of leaves that were beaten with a small club ("mazzola"). This fibre was then twisted round itself, the other end being held firm under a foot, to obtain very long, thin ropes from which thicker ropes ("libàni") could be woven. These were then sold, for use either in mussel production or as ropes for vessels. Raw fibre was also used to stuff the small mattresses of the ships' crews. The use of *Ampelodesmos*, particularly as wattle, is cited by Zambardi and Iannacone [20]. Rispoli [21], describing the nearby Salerno province where the plant could be found, suggests also its industrial exploitation. *Ampelodesmos *leaves were also utilized e.g. to light fires and for making brooms and small brushes used for cleaning cinders from fireplaces; the small stem is called "jàccola", the name probably deriving from their being used to make torches (fiàccole) for travelling by night. A number of stems were tied together in small bunches to make the torch, a use also reported by Guarrera [9].

Another occupation that was once important but that has disappeared entirely today is charcoal production. Large amounts of firewood (up to 20^3^), were piled up to form cones about 2 m high and then covered first with *Ampelodesmos *or *Sambucus ebulus *leaves, and then with a layer of about 10 cm of earth, after which the fire was lit through an opening at the base. Combustion lasted for several days and one could be sure that the process was finished when, making holes at the bottom of the heap, greenish smoke no longer emerged. Brief mention of charcoal production is made by Lieutaghi [13] for *Fagus sylvatica*, *Quercus ilex, Q. petraea *and *Q. robur*; by Pirone [14] for the mentioned species and for *Quercus cerris*.

The production of lime was another occupation today extinct. Lime was obtained by bringing as much as 500 quintals of calcareous rocks of the area to a very high temperature in special, large fireplaces built in hollows on steep slopes. The fuel consisted of the different shrubby species of the maquis and the undergrowth, which were cut, tied in big faggots and dried before being used. "Baking" took three days, during which combustion had to be uninterrupted, a task which involved burning up to 1500 faggots!

*Q. pubescens *wood was instead once the object of a flourishing activity producing railway sleepers, as well as roof and ceiling beams. Embers and cinders are removed from the breadoven by means of a small, long handled broom ("mùnnulu"), made from fresh branches of *Euphorbia characias, Laurus nobilis*, *Ruscus aculeatus *or *S. ebulus*. Elsewhere the "mùnnulu" was made with these plants or with *Ficus carica *and *Sorghum bicolor *[9].

Several plant species are employed in occupations related to sheep-rearing and cheese making. Shepherds crooks are generally made from shrubby species that provide a particularly hard wood [10]: *Prunus spinosa*, *Cornus mas *and *Crataegus monogyna*. Typical collars ("puntagliere", "collane") for goat bells, of excellent craftsmanship (Fig. 4) are made from *Acer neapolitanum*, *A. campestre *and *Juglans regia *wood.

In the absence of rennet, obtained from the stomach of kids or calves, milk could be curdled also with small, freshly gathered pieces of leafy branches of *F. carica *[9,22]; freshly-made cheeses are placed in special containers ("fuscelle","recuttari") woven, once again, from *Holoschoenus australis *and *Carex distans *stems.

The use of *Origanum heracleoticum *to dye wool red was previously unpublished (Lieutaghi [12] reports an analogous dye use for *Origanum vulgare*); the dye use of *Fraxinus ornus *cinder to obtain grey shades was commoner [23]. The husk of *J. regia *was still used to obtain brown-dark or black shades. Writing ink was prepared by squeezing the fruits of *S. ebulus*.

A most particular use was reported for a plant, which has now been a long-since naturalized species in Italy: *Agave americana*. The tough terminal prickle of the leaves and the tenacious fibres connected to it, removed together, were utilized as needle and thread for mending or sewing more or less unrefined fabrics. An analogous use is cited by Parada et al. [24]. "Spartu" (*Spartium junceum*) was, instead, also used for making clothes, in addition to flax. The fibre was extracted by beating and then washing small bunches of the previously boiled flexible stems (for fibre extraction see also Musacchio and Barone Lumaga [25]). This activity was carried out in the territory under study by a few families, while in other areas of southern Italy [26], and above all in Calabria, it appears to have flourished notably during the 1930s in the fascist period. During this era an autocratic economy producing food and clothes (e.g. with *Spartium junceum *fibers) without foreign imports existed in Italy. With the inflorescences of *A. donax *and *Arundo pliniana *particularly good brooms were made. Another type of small broom used after threshing to separate the grain of *Triticum aestivum *from the bran was made with *Calamintha nepeta *stems and leaves; this particular use is previously unpublished. A typical use in this area, but elsewhere less frequent [9] was that of flavouring home-made soaps with *Salvia officinalis*.

The use of *Euphorbia dendroides *latex once poured into streams to stun and, therefore to more easily catch fishes and eels is known also in other areas [15,26-29]. In the past, during the kill of the pig, a tool named "gammieddu" was used, made with *Phyllirea latifolia *or *Olea europaea *wood. This tool was made selecting a branch of about a meter with a shape similar to that of a boomerang; its tips were sharpened and endowed with cuts for the tendons of the hind legs of the pig; in this way the body of the pig could be hoisted, making dissection more easy.

## Conclusion

Data shows that practically all agricultural uses are still practised (except for "sovescio", green manure) and that almost half domestic-handicraft uses are still presently used. The differences between present and past uses in the cited categories: e.g. ropes, textile fibres and tobacco substitutes are all past uses, while walking sticks and fuel are current uses; collars, baskets and racks, brooms are partly made also today. The rediscovery of the folk uses of plants in the area under consideration is not only of historical and scientific value, but could also represent future, economic potential for the area. Several plants could still today be involved in the production of typical and appealing artefacts. In particular, the production of typical objects that are now on the decline (collars, baskets, clothes of particular textile fibres, and generally the artefacts under sale) could regain importance in the local economy.

## Supplementary Material

Additional file 1 Table 1 - Agricultural and domestic-handicraft uses of plants in the Tyrrhenian sector of Basilicata. Click here for file
