# Supplementary material for: Agricultural, domestic and handicraft folk uses of plants in the Tyrrhenian sector of Basilicata (Italy)
Source: J Ethnobiol Ethnomed. 2005 Jul 29;1:2. doi: 10.1186/1746-4269-1-2 (PMC1266048; doi:10.1186/1746-4269-1-2)
Supplement: Additional file 1 — Table 1 - Agricultural and domestic-handicraft uses of plants in the Tyrrhenian sector of Basilicata. [file 1746-4269-1-2-S1.doc]

Table 1 – Agricultural and domestic-handicraft uses of plants in the Tyrrhenian sector of Basilicata

| Family | Scientific and local name  (voucher specimen) | Habi-  tat | Parts  used | Popular uses, function and citations | Fre-  quency | Users | Use |
| --- | --- | --- | --- | --- | --- | --- | --- |
| Pterydophyta |  |  |  |  |  |  |  |
| Hypolepidaceae | *Pteridium aquilinum* | Sbu | Aerial parts | Litter for domestic animals ° (4) | C | P,F,O | P |
|  | (L.) Kuhn [filici] S1 |  | Aerial parts | Brooms to clean ovens from ashes and sticks*°* * (4) | C | P,F,O | P |
|  |  |  | Aerial parts | Apples stored between layers of leaves over  the winter * (1) | R | O | P |
| **Angiospermae**  **Dicotyledones** |  |  |  |  |  |  |  |
| Aceraceae | *Acer neapolitanum* Ten. | Wo | Branches | Baskets ° * (2) | R | O | P,S |
|  | [Acero] S34 |  | Branches | “Collane” and “puntagliere” (collars with bells for cows) *°* * (3) | C | P,O | P |
|  |  |  | Wood | Fuel in the fireplace ° * (2) | C | P,F,O | P,S |
|  |  |  | Wood | Furniture and coffins * (1) | Vc | O | P,S |
|  |  |  | Wood | Soles of footwear “zoccoli” (clogs) * (1) | R | F,O | P,S |
|  | *Acer campestre* L. [Occhiu] S35 | Wo | Wood | Handles for tools (3) | R | F,O | P,S |
|  |  |  | Branches | “Collane” and “puntagliere” (collars with bells for cows) *°* (3) | C | P,O | P |
|  |  |  | Wood | Yokes (“jugo”) for oxen during ploughing (2) | C | O | P,S |
| Anacardiaceae | *Pistacia lentiscus* L. [Lentisco] S32 | Mmq | Small branches | Funeral wreaths * (2) | Vc | O | P |
|  | *Pistacia terebinthus* L.. [Tràmmisi] S33 | Wo | Wood | “Ciaramella” (musical instrument) and bagpipe (1) | R | O | P,S |
| Asteraceae | *Inula viscosa* (L.) Aiton [Crisi] S51 | Unc | Branches,  Leaves | Brooms repellent for fleas * (2) | R | F,O | P |
|  | *Matricaria chamomilla* L. [Cambumilla] S52 | Kiga | Flowery tops | In wardrobes and drawers to keep moths away *°* (3) | C | O | P |
|  |  |  | Flowery tops | Bunches as a bee repellent (1) | R | O | P |
| Betulaceae | *Alnus cordata* (Loisel.) Desf. | Wo | Wood | Fuel for the hearth ° (4) | Vc | P,F,O | P,S |
|  | [Verna bianca] S6 |  | Wood | Fuel in ovens, for baking bread ° (2) | Vc | P,F,O | P,S |
|  |  |  | Wood | Wood sold for making boxes for fruit ° (3) | R | O | S |
|  |  |  | Wood | Furniture and coffins (1) | Vc | O | S |
|  |  |  | Dried branches | Training climbing pea plants ° * (3) | C | P,F,O | P |
|  |  |  | Wood | Soles of footwear called “zoccoli” (clogs) (1) | R | F,O | P,S |
|  | *Alnus glutinosa* (L.) Gaertner | Wo | Wood | Fuel in ovens, for baking bread ° (2) | Vc | P,F,O | P,S |
|  | [Verna gialla] S7 |  | Wood | Wood sold for making boxes for fruit (2) | R | O | S |
|  |  |  | Wood | Hollowed trunks as mangers “stagliate” (2) | R | F,O | S |
|  |  |  | Wood | Spokes of cartwheels of carts (“traini”) (1) | R | O | P |
| Caprifoliaceae | *Sambucus ebulus* L. [Iévulu] S49 | Ant | Flowery tops | Juice used as ink for writing (2) | R | P,F,O | P |
|  |  |  | Stems, leaves | Insulation between earth and wood to create charcoal * (2) | C | P,F,O | P |
|  |  |  | Stems, leaves | Brooms for cleaning wood-burning stoves (1) | C | P,F,O | P |
|  | *Sambucus nigra* L. [Sàmmucu] S50 | Ant | Stems | Whistles * (1) | C | F,O | P |
| Cornaceae | *Cornus mas* L. [Sanginello] S38 | Wo | Branches | Walking sticks for shepherds ° (1) | C | O | P |
|  |  |  | Branches | Brooms for sweeping open spaces * (2) | R | P,F,O | P |
|  |  |  | Branches | Baskets (1) | R | O | P,S |
| Corylaceae | *Carpinus orientalis* Miller [Carpino] S8 | Wo | Branches | Training plants and fencepoles ° * (3) | R | P,F,O | P |
|  | *Corylus avellana* L. [Nocella] S9 | Wo | Branches | Baskets ° (1) | C | P,F,O | P,S |
|  | *Ostrya carpinifolia* Scop.  [Carpino scuro] S10 | Wo | Branches  Wood | Training plants; fencepoles *°* (3)  Fuel for the fireplace *°* (2) | C/R  Vc | P,F,O P,F,O | P  P,S |
| Ericaceae | *Erica arborea* L. [Elica masculina] S39 | Ga | Branches | Brooms for rooms (6) | C | F,O | P |
|  | *Erica multiflora* L. [Elica femminina] S40 | Ga | Branches | Brooms for rooms (4) | C | F,O | P |
| Euphorbiaceae | *Euphorbia characias* L.[Pede de lupo] S30 | Ro | Stems, leaves | Brooms to clean ovens from ash, sticks *°* (3) | Vc | F,O | P |
|  | Euphorbia dendroides L. [Tasso, rogna] S31 |  | Latex | Put in streams to stun and capture eels (2) | R | P,F,O | P |
| Fagaceae | *Castanea sativa* Miller | Wo | Branches | Baskets ° (4) | Vc | P,F,O | P,S |
|  | [Castagno] S11 |  | Wood | Barrels (wine production) *°* (5) | R | P,F,O | P,S |
|  |  |  | Wood | Poles to support plants (“tutori”) *°* (5) | Vc | P,F,O | P,S |
|  |  |  | Wood | Furniture (6) | C | P,F,O | P,S |
|  |  |  | Branches | Training plants and as fencepoles ° (7) | Vc | P,F,O | P,S |
|  |  |  | Branches | Beams for ceilings and rooves (3) | Vc | P,F,O | P,S |
|  | *Quercus cerris* L. [Cerro] S12 | Wo | Wood | Barrels (wine production) *°* (2) | R | P,F,O | P,S |
|  |  |  | Wood | Fuel for hearths *°* (2) | Vc | P,F,O | P,S |
|  | *Quercus ilex* L. [Ilece] S13 | Wo | Wood | Handles for tools ° (12) | Vc | P,F,O | P,S |
|  |  |  | Wood | Hammer (“mazza”) to split wood ° (3) | C | P,F,O | P |
|  |  |  | Wood | Fuel in ovens and in the fireplace *°* (2) | Vc | P,F,O | P,S |
|  |  |  | Ashes | Detergent for clothes (“lissìa”) * (1) | C | F,O | P |
|  |  |  | Branches,  leaves | Screens to protect citrus trees from frost *°* * (3) | Vc | P,F,O | P |
|  | *Quercus pubescens* Willd. | Wo | Wood | Fuel in the fireplace *°* (5) | Vc | F,O | P,S |
|  | [Cersa] S14 |  | Wood | Handles for tools *°* (2) | Vc | P,F,O | P |
|  |  |  | Wood | Railway sleepers and beams for ceiling and roves (4) | C | P,O | S |
|  |  |  | Wood | Lintels for windows and doors (2) | C | O | P,S |
|  |  |  | Wood | Barrels (wine production)*°* (3) | R | P,F,O | P,S |
|  |  |  | Wood | Trunks split along the length to train vines *°* (3) | C | F,O | P |
|  |  |  | Wood | Collars with bells for cows and goats (3) | R | O | P,S |
| Juglandaceae | *Juglans regia* L. [Noce] S5 | Cu | Husk | Decoction for dyeing dresses black (7) | C | F,O | P |
|  |  |  | Husk | Decoction applied to the hair (hair dye) (2) | R | O | P |
|  |  |  | Wood | Furniture (4) | C | F,O | P,S |
|  |  |  | Wood | Ploughs (1) | C | F,O | P,S |
|  |  |  | Wood | Collars for cows and goats (3) | R | O | P,S |
|  |  |  | Wood | Soles of footwear called “zoccoli” (clogs) (1) | R | F,O | P,S |
| Lamiaceae | *Calamintha nepeta* (L.) Savi [Nepeta] S44 | Unc | Aerial parts | Small brooms to separate chaff from grain  after threshing * (3) | C | P,F,O | P |
|  |  |  | Aerial parts | Rubbed onto pots and pans (detergent) *°* * (2) | Vc | F | P |
|  | *Lavandula angustifolia* Miller [Spica, Spicaddosa] S45 | Ga | Flowery tops | To perfume drawers and cupboards *°* (3) | C | O | P |
|  |  |  | Flowery tops | Tops to perfume home-made soap * (3) | R | F,O | P |
|  | *Origanum heracleoticum* L. [Rìgono] S46 | Sua | Flowery tops | To dye wool red * (1) | R | P,F,O | P |
|  | *Salvia officinalis* L. [Salvia] S47 | Ga | Leaves | Leaves to perfume home-made soap (2) | R | O | P |
|  |  |  | Dried leaves | As a tobacco substitute * (2) | R | P,O | P |
| Lauraceae | *Laurus nobilis* L. [Lauro] S20 | Da | Stems,  leaves | Brooms to clean ovens from ashes (2) | C | P,F,O | P |
| Leguminosae | *Lathyrus sylvestris* L. [Oleca] S25 | Fal | Entire plant | Green manure (‘sovescio’) * (1) | C | F,O | P |
|  | *Robinia pseudacacia* L. [Càggia] S26 | Unc | Wood | Handles for tools ° (1) | R | P,F,O | P,S |
|  | *Spartium junceum* L. [Spartu] S27 | Sua | Stems | Textile fibres (10) | R | F,O | P,S |
|  |  |  | Stems | Edges of racks for drying foodstuffs *°* (8) | C | P,F,O | P,S |
|  |  |  | Branches | Screens to protect citrus trees from frost (4) | C | P,F,O | P |
|  |  |  | Stems | Twine for plants (to poles) *°* (6) | Vc | P,F,O | P |
|  |  |  | Stems | Twine for young vine stems ° (3) | Vc | P,F,O | P |
|  |  |  | Stems | Containers used for drying cheeses (4) | C | F,O | P,S |
|  |  |  | Stems | Brooms to sweep open spaces * (2) | C | P,F,O | P |
|  | *Vicia* sp.pl. [Vezze] S28 | Ocu | Entire plant | Green manure (‘sovescio’) * (2) | C | F,O | P |
| Linaceae | *Linum usitatissimum* L. [Lino] S29 | Ocu | Stems | Textile fibres (3) | R | O | P |
| Moraceae | *Ficus carica* L. [Ficàra] S16 | Cu | Wood | Green twigs to curdle milk (4) | R | O | P |
| Myrtaceae | *Myrtus communis* L. | Mmq | Aerial parts | Fuel to produce lime from limestone * (4) | C | P,F,O | S |
|  | [Murtìdda] S37 |  | Branches | Brooms for sweeping open spaces * (5) | C | P,F,O | P |
|  |  |  | Stems, leaves | Brooms to clean wood-burning stoves ° * (1) | R | F,O | P |
|  |  |  | Branches | Baskets * (1) | R | O | P,S |
|  |  |  | Leaves | For tanning leather (3) | C | P,F,O | S |
|  |  |  | Stems, leaves | Funeral wreaths ° * (1) | C | O | S |
| Oleaceae | *Fraxinus ornus* L. [Milegro] | Wo | Branches | Baskets ° (9) | Vc | P,F,O | P,S |
|  | S41 |  | Branches | Large baskets (“coffòni”) for the transport  by ass *°* (2) | Vc | P,F,O | P,S |
|  |  |  | Wood | Ploughs (4) | C | F,O | P,S |
|  |  |  | Branches | Handles for scythes and other tools (5) | R | F,O | P,S |
|  |  |  | Branches | “Collane” (collars with small bells) for cows and goats ° (4) | R | O | P,S |
|  |  |  | Branches, Wood | Walking sticks and poles for plants *°* * (3) | C | F,O | P |
|  |  |  | Bark | Dye (decoction for cotton cloth) (2) | C | P,F,O | P |
|  | *Olea europaea* L. [Olivo] S42 | Cu | Small branches | Baskets ° (1) | Vc | O | P,S |
|  |  |  | Branches | Sticks mainly used by shepherds *°* (3) | C | P,F,O | P |
|  |  |  | Branches | Tool to hang the pig during slaughter (2) | R | P,F,O | P |
|  |  |  | Wood, banches | Fuel for the fireplace ° (3) | C | P,F,O | P |
|  | *Phyllirea latifolia* L. [Agròmmeto] S43 | Mmq | Small branches | To attract flies at the entrance to cowsheds ° * (1) | C | P,F,O | P |
|  |  |  | Branches, Wood | Walking sticks and poles for plants *°* * (1) | C | P,F,O | P |
|  |  |  | Branches | “Collane” and “puntagliere”, collars with bells for cows (1) | R | P,O | P |
|  |  |  | Branches | Tool to hang the pig during slaughter (2) | R | P,F,O | P |
| Ranunculaceae | *Clematis vitalba* L. [Turtagne] S19 | He | Stems | Dried stems as a tobacco substitute (3) | C | P,O | P |
| Rosaceae | *Crataegus monogyna* Jacq. | Bu | Wood | Implement (“rèmunu”) used in making cheese *°* (1) | C | P | P |
|  | [Spina cerasola, Spina di |  | Branches | Walking sticks used by shepherds *°* (1) | C | P,F,O | P |
|  | pirune] S21 |  | Wood | Fuel for the fireplace *°* (2) | C | P,F,O | P |
|  | *Prunus avium* L. [Ciliegio] S22 | Cu | Wood | “Tini” (wine production) and furniture (2) | C | P,F,O | P,S |
|  |  |  | Wood | Blocks to repair the soles of footwear (1) | R | F,O | P,S |
|  | *Prunus spinosa* L. [Prumma selvaggia] S23 | Bu | Branches | Walking sticks used by shepherds ° (2) | C | P,F,O | P |
|  | *Pyrus amygdaliformis* Vill. [Pirànio] S24 | Sbu | Entire plant | Graftings ° (6) | C | F,O | P |
| Salicaceae | *Salix alba* L. subsp. *vitellina* (L.) | Ecu | Branches | Twine for vines *°* (6) | Vc | P,F,O | P |
|  | Arcang. [Salice] S2 |  | Branches | Sieve for cereals or pulses (1) | C | F,O | P,S |
|  | *Salix purpurea* L. [Vignarello] S3 | Ri | Branches | Baskets ° (7) | Vc | O | P,S |
| Santalaceae | *Osyris alba* L. [Scannagaddine] S4 | Wo | Branches | Brooms for sweeping rooms ° (2) | C | P,F,O | P,S |
| Solanaceae | *Solanum tuberosum* L. [Patata] S48 | Cu | Dried leaves | As a tobacco substitute * (1) | R | P,O | P |
| Ulmaceae | *Ulmus minor* Miller [Urmo] S15 | Wo | Wood | Handles for tools (3) | C | F,O | P,S |
|  |  |  | Wood | Planks used in carpentry (1) | R | O | P,S |
|  |  |  | Wood | Training plants and as fencepoles ° (2) | C | F,O | P |
| Urticaceae | *Parietaria diffusa* M. et K. S17  *Parietaria officinalis* L. [Mentosa] S18 | Wa | Aerial parts | Rubbed onto pots and pans (detergent) *°* (7) | Vc | F | P |
| Vitaceae | *Vitis vinifera* L. [Vite] S36 |  | Branches | Dried bark as a tobacco substitute (3) | C | P,O | P |
|  |  |  | Leaves | Protection for cottage cheese from insects/ dust (1) | R | P,F, O | P |
| **Angiospermae**  **Monocotyledones** |  |  |  |  |  |  |  |
| Agavaceae | *Agave americana* L. [Semprevivi] S54 | Unc | Leaves | Last spike/fibre as needle and thread for  sewing or mending * (4) | R | F | P |
| Cyperaceae | *Carex distans* L. [Juncu] S59 | Da | Stems | Moulds for cheese (“fuscedde”) *°* * (3) | C | P,F,O | P,S |
|  | *Holoschoenus australis* (L.) | Da | Stems | Moulds for cheese (“fuscedde”) *°* * (12) | Vc | P,F,O | P,S |
|  | Reichenb. [Juncu] S60 |  | Stems | Moulds for cottage cheese (“recottaro”) *°* * (12) | Vc | P,F,O | P,S |
|  |  |  | Leaves | Twine for climbing vegetables ° * (3) | C | P,F,O | P |
| Liliaceae | *Ruscus aculeatus* L. [Rùsciuli] | Wo | Stems | To keep mice away ° (9) | C | F,O | P |
|  | S53 |  | Stems | Brooms for cleaning wood-burning ovens ° (5) | Vc | F,O | P |
|  |  |  | Stems | Small brooms for cleaning chimneys ° (2) | C | F,O | P |
| Poaceae | *Ampelodesmos mauritanicus* | Ga | Leaves | Rope used in cultivating mussels * (10) | Vc | P,F,O | P,S |
|  | (Poiret) T. Durand et Sch. |  | Leaves | Rope for boats and in several activities * (12) | Vc | P,F,O | P,S |
|  | [Tagliamani, Erba alfa, Jaccole (stems)] S55 |  | Stems | Racks (“grate”, “cestedde”) to dry cheese,  home-made pasta, fruit and vegetables *°* * (7) | Vc | F,O | P,S |
|  |  |  | Stems | Cages (“gratedde”) for capturing birds * (5) | C | P,F,O | P |
|  |  |  | Stems | Edges of sieves (see *Salix alba*) * (1) | C | F,O | P,S |
|  |  |  | Leaves | Fibre to stuff mattresses used by sailors * (8) | C | O | S |
|  |  |  | Leaves | Brooms to sweep farmyards, sheds, cellars (7) | C | P,F,O | P,S |
|  |  |  | Leaves | For mangers (bedding for cattlesheds) * (3) | C | P,F,O | P |
|  |  |  | Stems, leaves | Fuel for starting fires ° * (4) | Vc | P,F,O | P |
|  |  |  | Leaves | Charcoal (see *Sambucus ebulus*) * (2) | C | P,F,O | P |
|  |  |  | Leaves | Twine for vegetables, vines, etc. *°* (9) | Vc | P,F,O | P,S |
|  |  |  | Leaves | Brushes for painting * (2) | R | O | P,S |
|  |  |  | Stems | Torches for travelling by night (2) | Vc | F,O | P |
|  |  |  | Leaves | Twine (“torta”) for bundles of wood and grass ° (3) | Vc | P,F,O | P |
|  | *Arundo donax* L. [Canna] S56 | Da | Entire plant | Brooms for sweeping rooms* (3) | R | F,O | P |
|  |  |  | Stems | Baskets (one type is called “coffe”) *°* (13) | C | F,O | P,S |
|  |  |  | Stems | Racks (see *A. mauritanicus*, “grate”) *°* (4) | Vc | F,O | P,S |
|  |  |  | Stems, leaves | Screens to protect citrus trees from frost ° * (1) | Vc | P,F,O | P |
|  |  |  | Stems | Training vines and vegetables ° (5) | Vc | P,F,O | P |
|  |  |  | Stems | Fixed one to other as support for plastering  ceilings * (2) | C | F,O | P |
|  |  |  | Stems | Strips for spreading smoked bacon ° * (1) | R | P,F,O | P |
|  |  |  | Stems | Stems threaded into dried figs to create  packets (“skocche” ) for storing them * (3) | C | P,F,O | P |
|  |  |  | Stems | Piece of stem writtled into a flute (2) | R | O | P,S |
|  |  |  | Stems | Reed instrument (“raccola”) * (2) | C | P,F,O | P |
|  | *Arundo plinii* Turra [Cannuccia] S57 | Bu | Flowery tops | Brooms for sweeping rooms * (1) | C | P,F,O | P,S |
| Typhaceae | *Typha latifolia* L. [Vùda] S58 | Da | Leaves | Seats of chairs * (3) | R | O | P,S |

**Habitat:** Ant = anthropic areas; Bu = bushes; Cu = cultivated areas; Da = damp areas; Ecu = edges of cultivated areas; Fal = fallow land; Fi = fields; Ga = garrigues; Kiga = kitchen gardens; H = hedges; Ma = mountain areas; Mmq = Mediterranean maquis; Mq = maquis; Ocu = once cultivated areas; Ri = rivers; Ro = rocks; Sbu = small bushes; Su = sunny areas; Unc =uncultivated areas; Und = undergrowth; Wa = walls; Wo = woods **Users:** P= personal; F= familiar; O= cited by others **Frequency:**  c= common; vc= very common; r= rare **Use:** p = private; s = sale

° = Current use  ***=** Not present in the consulted scientific literature (Beconcini et al., 1984; Corsi et al. 1980; Fenaroli 1967; Guarrera 1994; Guarrera, 2000; Lentini e Raimondo, 1990; Lieutaghi, 1974; Lieutaghi, 1975; Pirone 1995; Raimondo e Lentini, 1990; Sella 1992)
